# Optimization of Microstructured Silicon Attenuated Total Reflectance Fourier Transform Infrared Elements

**DOI:** 10.1021/acs.analchem.6c02804

**Published:** 2026-08-27

**Authors:** Eleanor L. Osborne, Aneesh Vincent Veluthandath, Waseem Ahmed, Ganapathy Senthil Murugan

**Affiliations:** † Optoelectronics Research Centre, 7423 University of Southampton, University Road, Southampton, SO17 1BJ, United Kingdom

## Abstract

Microstructured silicon attenuated total reflection (ATR) Fourier transform infrared (FTIR) elements, commonly known as microstructured silicon reflection elements (μSREs), are gaining popularity as a low-cost, disposable alternative to ATR crystals in fields such as medical diagnostics. They comprise a silicon chip, with light incident on microprisms on the underside guided into the chip and totally internally reflecting at the sample interface. However, such devices are prone to issues, including poor limit of detection compared to traditional ATR crystals, and fringes within their spectra, whose origin has not been conclusively identified in the literature. This work, verified by experimental data, mathematically reveals the origin of these fringes to be an acquired phase difference between beams incident on the same μSRE prism face upon input, but exiting the μSRE through separate prism faces, resulting in interference. This improved understanding of the behavior of light within the μSRE will enable researchers to select appropriate μSRE designs for their application, maximizing the potential of this technology.

## Graphical abstract

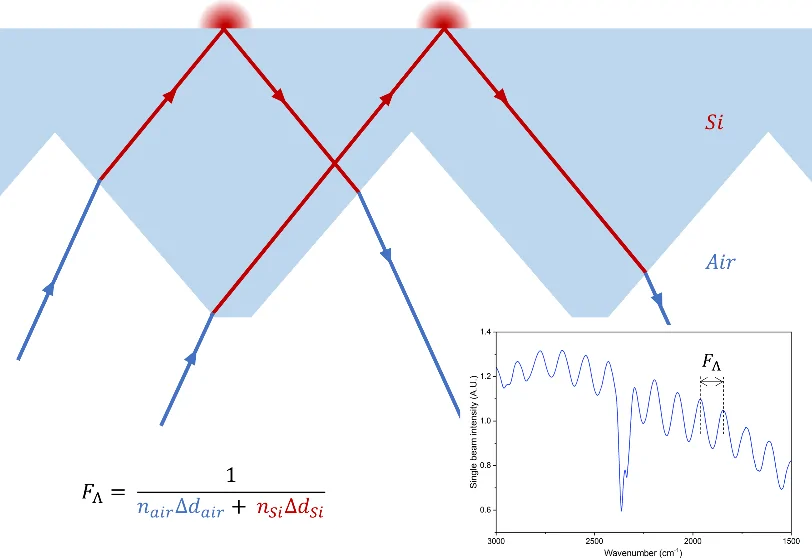

## Introduction

Fourier transform infrared (FTIR) spectroscopy is a vibrational spectroscopic technique that provides label-free, highly specific, quantitative data about a sample through the generation of a “fingerprint” spectrum. However, standard transmission measurements suffer from signal saturation when measuring highly absorbing samples, particularly if dissolved in water. As such, the attenuated total reflectance (ATR)-FTIR variant may be used, whereby only the evanescent field of a beam totally internally reflecting at the interface of a crystal with a lower refractive index sample on top, may be used to overcome such a shortcoming. However, ATR crystals tend to be high cost, and prone to scratching in the case of ZnSe, making them of limited use in a setting such as medical diagnostics, where ease of cleaning or single use is key.

Thus, microstructured silicon ATR-FTIR reflection elements (μSREs), also called microstructured silicon internal reflection elements, were developed by Schumacher et al.^1^ in 2010, as low cost-disposable alternatives to replace standard ATR crystals. μSREs comprise of prisms etched on the underside of a silicon chip, as shown in Figure 1. Prism sidewall angle β is defined by the crystal plane, with β = 35.3° for (100) silicon (Si) and β = 54.7° for (110) Si. Light incident on the prism at elevation angle of incidence θ_*i*_ refracts, and subsequently totally internally reflects at the sample−μSRE interface with angle γ. An evanescent field extends into the sample, whose penetration depth is dependent on γ. In addition, the μSRE may be rotated to change the azimuthal angle of incidence ϕ; ϕ = 0° corresponds to light incident parallel to the axis of the prisms, while ϕ = 90° corresponds to light incident perpendicular to the axis of the prisms.

**Figure 1:**
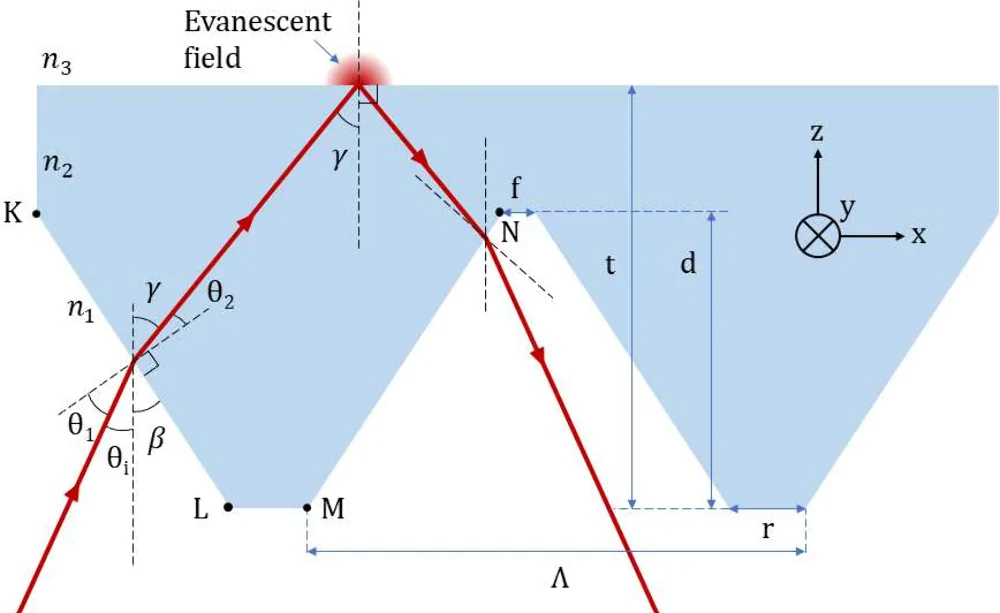
Diagram of a cross-section of a Si μSRE with light incident with angle θ_*i*_ and ϕ = 90°. β is the angle of the prism sidewall, γ the total internal reflection angle, Λ the pitch of the prisms, *d* is prism etch depth, *r* the prism roof, *f* is the flat at the base of the prism if etching is not fully performed, *t* is the wafer thickness, *n_1_*, *n*_2_ and *n_3_* are the refractive indices of air, silicon and the sample, respectively, θ_1_ is the incident angle and θ_2_ is the refraction angle.

Several applications of such devices have been demonstrated over recent years, including nitrate sensing in water,^2^ triaging of brain cancer,^3,4^ chemical imaging of microfluidic cells,^5^ electrochemical ATR-SEIRAS^6^ and additional enhancement through the inclusion of subwavelength structures on the topside of the μSRE.^7^ The reader is referred to the review by Burgess et al.^8^ for a full discussion of recent uses of this technology. Several commercially available devices are available, including the X,Y Autosampler from PIKE Technologies, Arrow from Specac, Dxcover blood analysis system, and IRUBIS.

Understanding of these devices is still developing. Deng et al.^9^ performed an analysis of commercially available μSREs, comparing the sensitivity, LOD and noise for IRUBIS, Dxcover and Specac Arrow devices. However, they identified fringes within the spectra whose origins could not be confirmed. Such fringes may cause issues in extracting quantitative data about a sample, so their minimization or removal is vital for extending the uptake of such μSREs. In this paper, we compare μSREs with varying prism dimensions and present a mathematical model for the source of these fringes, enabling researchers to use μSREs optimized to their intended application.

## Methodology

### Fabrication

μSREs of varying parameters (shown in Table 1) were fabricated using (110) double-side polished 6” silicon wafers, using the methodology shown in Figure 2. 1.3 μm thermal oxide was grown using wet oxidation. There were two rounds of photolithography performed in this fabrication; the first was used to establish the crystal plane orientation, the second to generate the underside prism features aligned to the ⟨112⟩ direction. The wafers were supplied with a single flat oriented to the ⟨110⟩ direction. To ascertain the crystal lattice orientation, photolithography was performed using S1813 photoresist (MICROPOSIT^TM^ S1818 positive photoresist solution from Dow®Shipley - Rohm and Haas Co.) spun at 2k rpm (thickness: ∼1.2 μm). A photomask with three circles (diameters 25, 50, and 100 μm) at the edge of the wafer was used.^10^ Inductively coupled plasma reactive ion etching (ICP-RIE) was used to etch the silica hard mask, then plasma ashing removed remaining photoresist. Seven minutes in 37% KOH at 80 °C produced an anisotropic etch of the crystal planes, enabling identification of their orientation.

**Figure 2:**
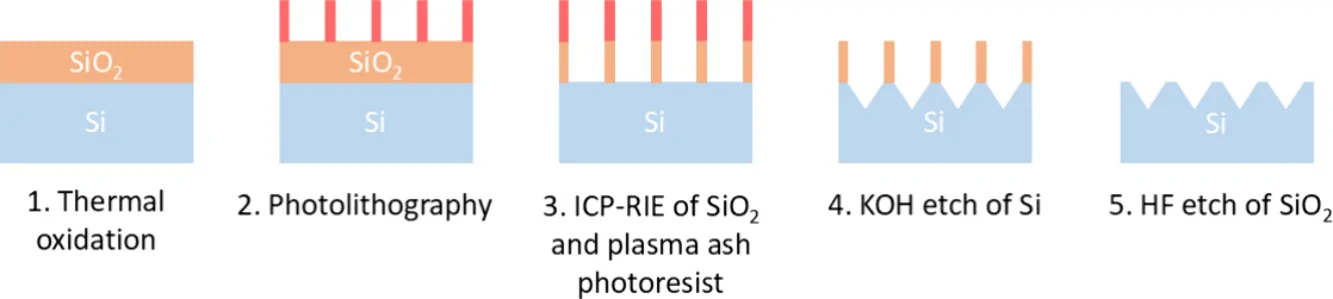
Fabrication methodology for microprisms etched on the underside of silicon chips. If plane orientation is to be determined, this is performed between steps 1 and 2.

**Table 1:** Dimensions of fabricated prisms^a^

number	Λ (μm)	*r* (μm)	*f* (μm)	*d* (μm)	*t* (μm)	β (°)	*A*
1	28	5	0	8	500	54.7	0.83
2	30	2	0	10	500	54.7	0.93
3	55	6	0	17	500	54.7	0.89
4	78	4	0	26	500	54.7	0.95
5	80	2	0	28	500	54.7	0.98
6	103	0	19	30	500	54.7	0.81
7	105	2	15	31	500	54.7	0.84
8	128	4	40	30	500	54.7	0.66
9	130	4	40	31	500	54.7	0.66
10	152	10	63	29	500	54.7	0.52
11	155	2	67	30	500	54.7	0.55
12	177	8	89	29	500	54.7	0.46
13	179	3	91	30	500	54.7	0.48
14	202	6	117	28	500	54.7	0.39
15	207	2	118	31	500	54.7	0.42
IRUBIS^5,9^	700	50	0	230	500	54.7	0.93
Specac Arrow	280	17	0	177	380	35.3	0.94
DXCover^11^	275	25	0	186	380	35.3	0.91

a Dimensions for Specac arrow chips were measured in house, while dimensions for IRUBIS, and DXCover chips are cited.

Subsequently, photolithography was performed on the wafers using S1813 resist spun at 2k rpm (thickness: ∼1.2 μm) to produce the prisms, with the axis of the prism aligned to the ⟨112⟩ direction. ICP-RIE was repeated to etch the silica mask, then KOH etching used to etch the silicon anisotropically for 14 min. Final etch depths *d* for each μSRE are shown in Table 1. Finally, an HF etch was used to remove remaining thermal oxide.

**Table 2:** Sensitivity (*S*) from Peak Height, RMS Noise (*N*), *S*/*N*, LOD and LOQ for μSREs 1, 4, and 12 Fabricated in This Work Using d-Glucose Solutions, Compared to the Values for Dxcover, Specac Arrow and IRUBIS Chips Calculated in Ref 9 ^a^

μSRE	sensitivity *S* (AU/mM)	RMS noise *N* (AU)	*S*/*N* (mM^–1^)	LOD (mM)	LOQ (mM)
1	3.94 × 10^–5^ ± 1.81 × 10^–6^	5.77 × 10^–5^	0.683	4.39	14.6
4	3.83 × 10^–5^ ± 1.82 × 10^–6^	5.92 × 10^–5^	0.647	4.64	15.5
12	2.14 × 10^–5^ ± 4.04 × 10^–7^	4.44 × 10^–5^	0.482	6.23	20.8
Dxcover^9^	2.44 × 10^–5^ ± 5.26 × 10^–7^	3.71 × 10^–5^	0.658	4.56	15.2
Specac arrow^9^	1.61 × 10^–5^ ± 2.07 × 10^–7^	7.29 × 10^–5^	0.221	13.6	45.3
IRUBIS^9^	7.01 × 10^–5^ ± 7.48 × 10^–9^	7.07 × 10^–6^	9.92	0.303	1.01

a All measurements were done with the μSRE in parallel configuration (ϕ = 0°), and unmasked. Measurements from ref 9 had an angle of incidence of 20°, whilst angle of incidence in this work was 40°, so the sensitivity of the commercially available chips should be higher due to greater evanescent field penetration depth.

### Spectral Acquisition

All characterization of μSREs was performed using an Agilent Cary 670 FTIR Spectrometer with a DTGS detector unless stated otherwise, nitrogen purging of the chamber, 32 scans and no polarizer. The μSREs were characterized using the PIKE Veemax III accessory, which provides capability to alter the value of θ_*i*_ between 30° and 80°, with the axis of the prisms parallel to the beam in the *x*–*y* plane unless otherwise stated. Eighteen single beam spectra were measured for each μSRE with resolution of 2 cm^–1^. For absorbance measurements, 50 μL of 500 mM sodium acetate (NaOAc) or d-glucose solution was pipetted onto the μSRE topside, with at least 9 spectra measured sequentially and backgrounded to water. Absorbance measurements were made with spectral range 6000–400 cm^–1^ and resolution 4 cm^–1^.

### Spectral Analysis

NaOAc spectra were corrected for water vapor absorption using the method described in Ahmed et al.,^12^ baselined using Peak Spectroscopy software, and averaged to determine absorbance of the 1550 cm^–1^ peak. d-Glucose spectra underwent the same process, with the 1033 cm^–1^ peak used. Sensitivity (*S*) was calculated as the slope of a linear fit to absorbance (*A*) versus concentration (*c*) data (*A* = *Sc*). Root mean square (RMS) noise (*N*) was extracted from spectra in the region 930–1150 cm^–1^. Limit of detection (LOD) was taken to be 3*N*/*S*, and limit of quantification (LOQ) 10*N*/*S*. Single beam spectra were corrected for water vapor absorption, averaged and smoothed using a Savitsky Golay filter (31 points, second order polynomial) before fringe period was extracted using a Fourier transform.

## Results and Discussion

### Prism Dimensions

Dimensions of prisms are shown in Table 1 measured using an optical microscope. *A* is defined as the fraction of the μSRE surface covered by prism sidewalls angled by β to the vertical as in eq 1:

1 $$A=1-\frac{r+f}{\Lambda }$$

Figure 3a shows the absorbance of 500 mM NaOAc as a function of *A*. It can clearly be seen that those μSREs with the largest values of *A* (and therefore smallest percentage area comprising horizontal faces such as LM) enable collection of spectra with the greatest absorbance. This is because the resulting value of γ for light incident on the roof/base (i.e., side LM) with angle of incidence between 0° and 90° is small (0° to 17°, respectively) which is subcritical. Thus, partial refraction and reflection occur at the μSRE-sample interface; the refracted beam may undergo multiple reflections or be scattered in the sample, resulting in minimal signal reaching the detector. The reflected beam may also undergo multiple total or partial reflections within the μSRE and refractions out of the μSRE, again significantly weakening any signal reaching the detector.

**Figure 3:**
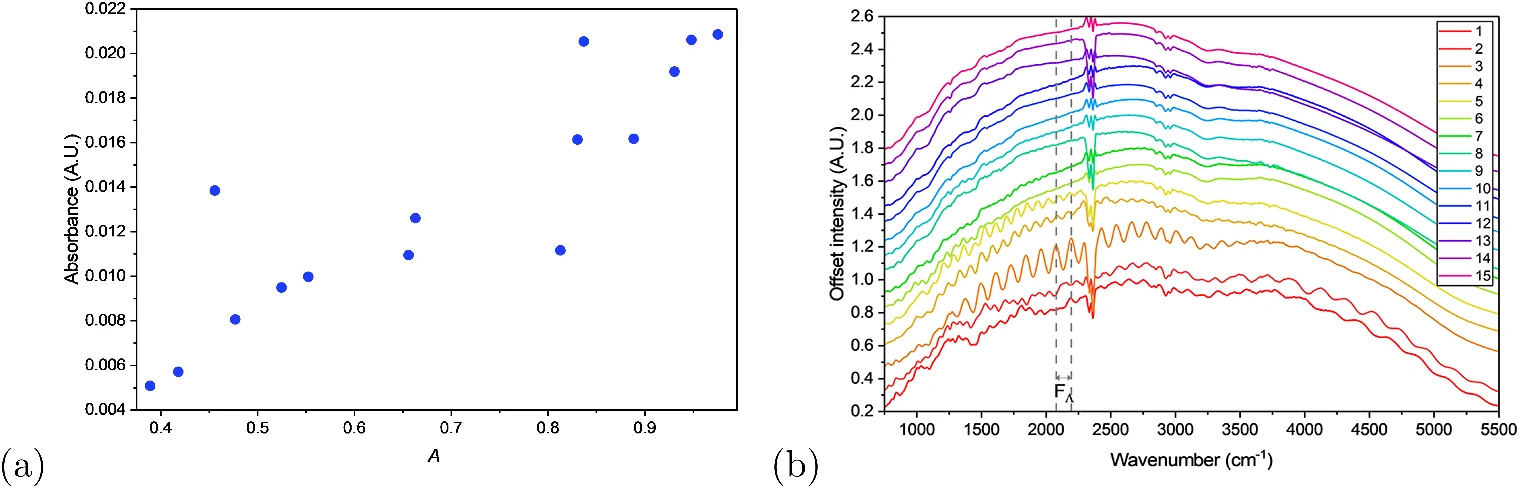
(a) Absorbance of 500 mM NaOAc backgrounded to water, measured on μSREs 1–15, plotted against the fraction of the μSRE surface covered by prism sidewalls angled by β to the vertical (*A*). (b) Normalized, offset single beam spectra of all μSREs, as numbered in Table 1. The original spectra are shown in Figure S6.

### Spectral Fringes

Figure 3b shows the single beam spectrum for μSRE 1–15; fringes are visible in some spectra, for example as denoted *F*_Λ_ for μSRE 3, with period varying between the spectra. It is notable that those prisms with larger pitch and larger roofs are less likely to produce visible fringes compared to prisms with a smaller pitch. However, these prism designs have smaller *A* values and therefore suffer as a consequence in absorbance as seen in Figure 3a. To ascertain the source of such fringes, a mathematical model was developed and its numerical model was established in Matlab; the details of this are discussed in Supporting Information. As shown in Figure 4a, assuming a plane wave, two rays incident on the underside of the μSRE on the same prism face may exit the μSRE on prism faces separated by one period. Such a phenomenon creates a path difference, which will lead to interference, with constructive interference occurring when the conditions of eq 2 are fulfilled:

2 $$m\lambda =n_{\mathrm{air}}\Delta d_{\mathrm{air}}+n_{\mathrm{Si}}\Delta d_{\mathrm{Si}}$$

where λ is the vacuum wavelength, m is an integer, *n*_air_ is the refractive index of the substance on the underside of the prisms (i.e., air), *n*_Si_ is the refractive index of the μSRE, and *d*_air,Si_ are the path lengths of the beams in each medium, respectively. Full details of the derivation of this mathematical model can be found in Supporting Information.

**Figure 4:**
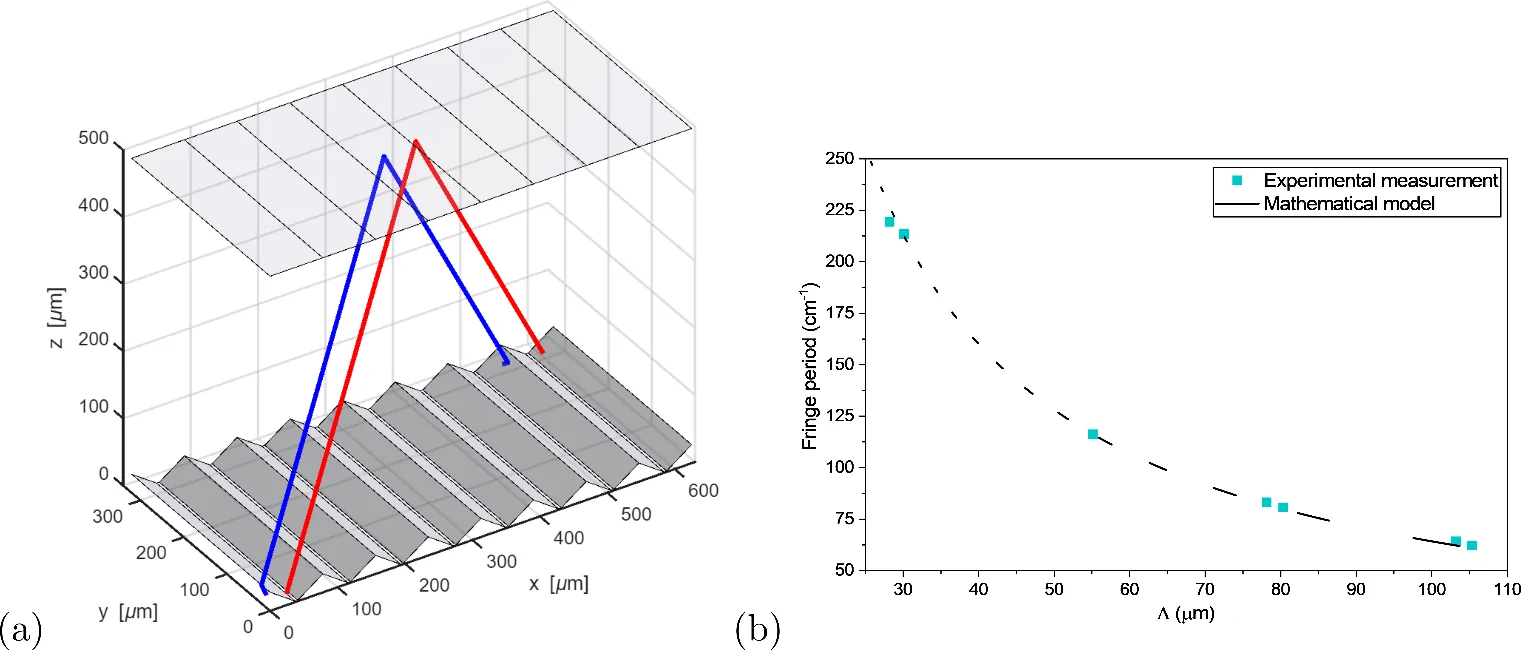
(a) Prism design 4 with rays incident at θ_*i*_ = 55° and ϕ = 0°. The two rays incident on a single prism plane exit the μSRE through separate prism planes, producing a path difference. (b) Fringe period calculated for varying prism pitch (θ_*i*_ = 40°, ϕ = 0°), plotted alongside experimentally measured fringe period for μSREs in Table 1. Missing regions of the lines indicate a prediction of no fringes occurring for such dimensions and angles.

To confirm this theory experimentally, the fringe period was extracted from the single beam spectra shown in Figure 3b using a Fourier transform of the entire spectrum, with these Fourier transforms available in the Supporting Information (Figure S6). These fringe periods are plotted in Figure 4b, alongside values predicted mathematically; the trend in values between the model and experiment agrees well.

The Fourier transform also produced fringe periods that were fractions of the fundamental fringe, as can be seen in Figure 3b for μSREs 1 and 2 in particular, and which can be observed in their spectra in Figure 3b at smaller wavenumbers. For μSREs 8–15, some fringes with a small amplitude can be discerned in Figure 3b, however these could not be distinguished from noise in their Fourier spectra (see Supporting Information Figure S3). For these μSREs, simulation showed that the size of the roof is such that the two beams cannot exit the μSRE from periodically adjacent prism faces, unless there is a large error in parameters such as incident angle, hence, the very weak fringe amplitude observed.

Using μSRE 4, the values of θ_*i*_ and ϕ were varied between 30–70° and 0–90° respectively, producing graphs in Figure 5a,b. There are clear fringes in these spectra, the periods of which were extracted using a Fourier transform (shown in Supporting Information, Figures S4 and S5), and plotted in Figure 5c,d. As θ_*i*_ decreases and ϕ increases, there is a trend toward increasing fringe period. Mathematically calculated fringe period is plotted in the same graphs, including variation in wafer thickness (*T*), prism pitch (Λ), *n*_Si_, ϕ and θ_*i*_ to account for realistic experimental error in such parameters. For example, Chan and Kazarian^13^ noted that the angle of incidence set on their Veemax II accessory did not align to the angle of incidence they estimated from measured effective thickness by as much as 5°. Gaps in the plotted lines for mathematical models indicate parameters at which fringes are not predicted. The model tends to underestimate fringe period by a few cm^–1^, however, accounting for potential experimental errors produces model values closer to those experimentally observed.

**Figure 5:**
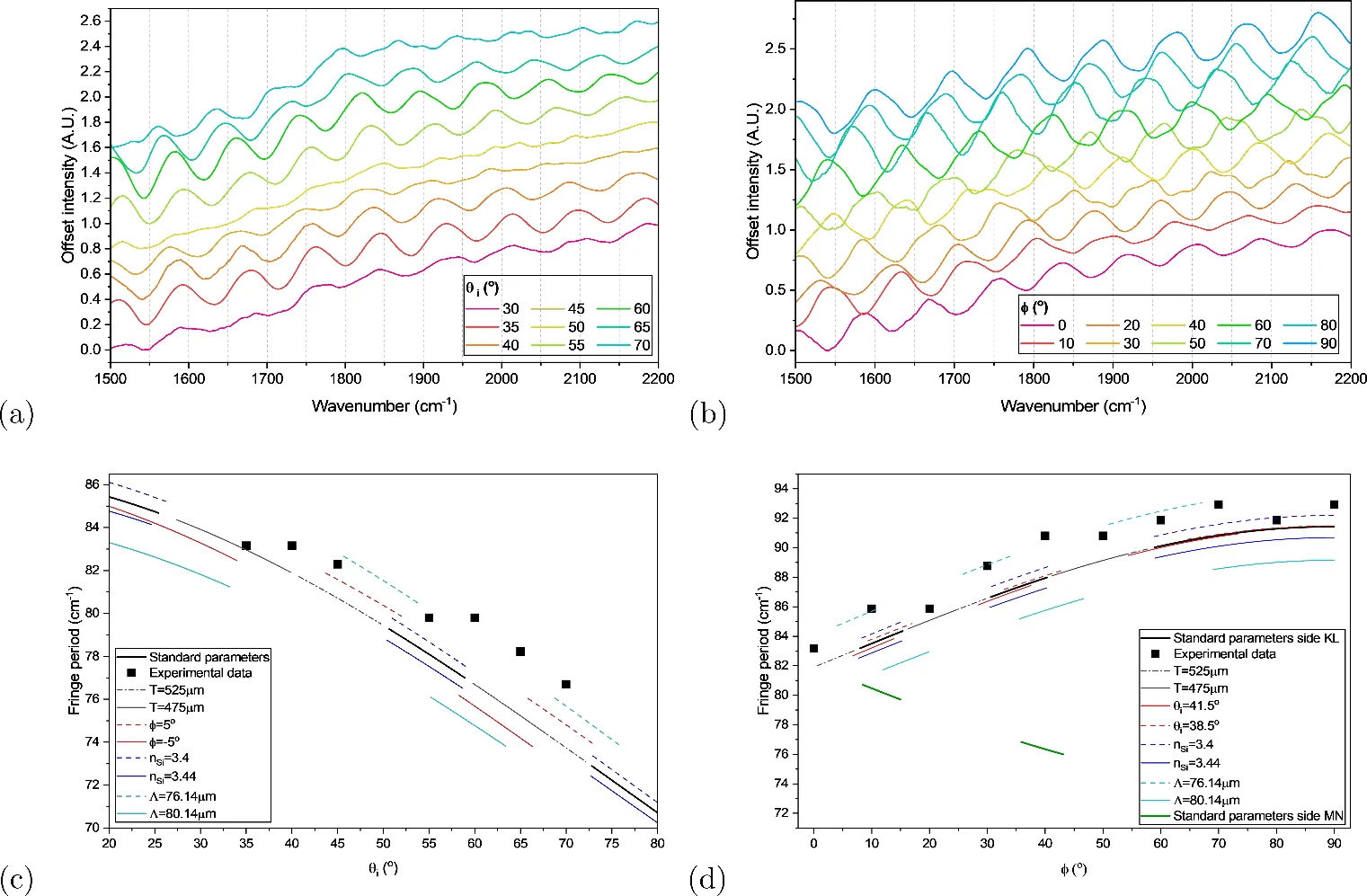
Offset intensity spectra for μSRE 4 in ATR configuration with (a) varying θ_*i*_ (ϕ = 0°), and (b) varying ϕ (θ_*i*_ = 40°). The fringe period was extracted from these intensity spectra, plotted for varying (c) θ_*i*_ (ϕ = 0°) and (d) ϕ (θ_*i*_ = 40°), alongside predicted fringe period calculated mathematically for a μSRE with dimensions of μSRE 4. In this case, *n*_Si_ was taken to be 3.42 for standard parameters. Wafer thickness *T*, prism pitch Λ, *n*_Si_, ϕ and θ_*i*_ were varied to establish how predicted fringe period varies with realistic experimental error or variation in these parameters. In (c) and (d), incidence is assumed to be on prism side KL unless otherwise stated.

It is important to note that, for values of ϕ ≠ 0°, the beam is incident on the two faces of a single prism (KL and MN in Figure 1) at different incident angles. Incidence on side MN may result in a value of γ that is subcritical, resulting in multiple internal reflections, and thus attenuation, within the μSRE. However, in the case where the resulting γ exceeds the critical angle, a path length difference resulting in spectral fringes may occur. However, in the Fast Fourier transform (FFT) of our spectra (Supporting Information, Figure S5), a single peak for fringe period corresponding to the beam incident on prism side KL is clearly visible, with other peaks far weaker. Figure 5d shows the calculated fringe period for a beam incident on prism side MN with standard parameters and ϕ varying. The predicted fringe period for sides KL and MN is closest when ϕ is smallest, while logically, the fraction of the beam interacting with side MN reduces with increasing ϕ, so it follows that the effects of such fringes are not easily distinguishable in the FFT in Figure S5. From this, we conclude that the dominant interference effects stem from the beam incident on side KL, and thus all results presented assume incidence on this side for simplicity. If fringes stemmed from the interference between the parts of the beam incident on KL and MN, it would be expected that no fringes would be observed for ϕ = 0° as no path difference occurs, but the observation of fringes (Figure 5a) proves this is not the case.

The model also predicted fringes of 16 cm^–1^ period for Specac arrow and Dxcover chips, and no fringes for IRUBIS chips at θ_*i*_ = 20° and ϕ = 0°, in agreement with Deng et al.’s^9^ experimental observations.

This work assumes a plane wave incident on the prisms with beam diameter equal to or greater than prism side KL. However, a standard FTIR spectrometer beam is convergent, so will contain be a distribution of incident angles, the effect of which we have explored in Figures 5c and d. This will result in a broader range of fringe periods visible in the FFT, as observed in the Supporting Information (Figures S4 and S5). However, a collimated beam (for example from a quantum cascade laser) would reduce this convergence issue. Furthermore, if the beam diameter is far smaller than the prism side KL, the beam may be input on a single prism face, with the likelihood of incidence on two faces separated by Λ upon output significantly reduced, resulting in substantial removal of fringes from the spectrum.

### Quantifying and Optimizing μSRE Performance

Upon inspection of how fringe period varies with prism pitch for differing incident angles (Figure 6a), it was noticeable that the range of prism pitch at which no fringes are predicted becomes wider as prism pitch increases until the prism dimensions become such that an incident beam will only interact with one prism. Furthermore, as the prism pitch increases, the fringe period decreases. Therefore, larger prisms have the advantage of the fringe period approaching the resolution of the spectrum also.

**Figure 6:**
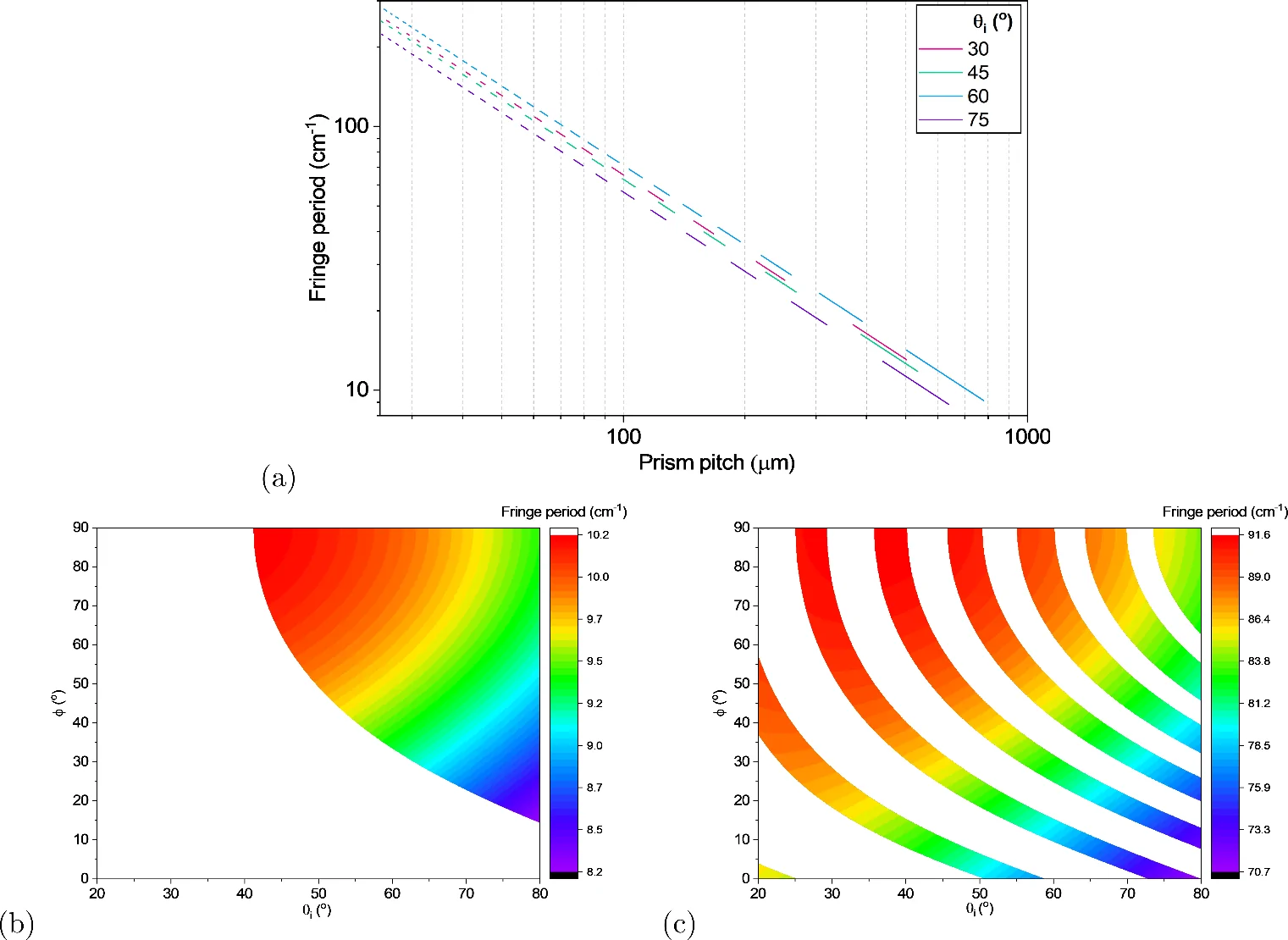
(a) Calculated fringe period for varying prism pitch, with incident angle ϕ = 0°. Calculated fringe period for varying angle of θ_*i*_ and ϕ for (b) the IRUBIS chip and (c) μSRE 4. The white regions indicate angles at which fringes are not predicted to occur due to this mechanism.

Figure 6 shows how the period of fringes calculated mathematically varies with incident angles θ_*i*_ and ϕ for (b) IRUBIS chips and (c) μSRE 4. The white regions indicate where no fringes are predicted through this mechanism, as the whole of the beam incident on one prism face is fully output on another prism face. It can be seen that IRUBIS chips are predicted to have fringes in their spectra only for large angles, while a large range of smaller angles produce no fringes, in agreement with the observations in ref 9. This means that any error in incident angles or prism dimensions should not impact the absence of fringes from the spectrum. It should be noted that IRUBIS recommend using their chips in the ϕ = 0° configuration only, with θ_*i*_ = 20°.^14^ On the other hand, μSRE 4 (Figure 6c) shows smaller spaces between regions in which no fringes are predicted. Therefore, any small variation in incident angle could result in fringes being present in the spectrum. This also poses an issue during measurement, where any small change in position of the μSRE between background and sample spectra could change the path difference condition, resulting in fringes in the sample spectrum.

In order to further quantify μSRE performance, serial dilutions of glucose ranging from 12.5 mM to 800 mM were measured on several μSREs (Figure 7), with incident angles θ_*i*_ = 40° and ϕ = 0°, enabling calculation of sensitivity (*S*), limit of detection (LOD), limit of quantification (LOQ) and noise (*N*), shown in Table 2. Similar measurements from ref 9 are also shown for Specac, Dxcover and IRUBIS chips, although the results are not directly comparable owing to the difference in incident angle (θ_*i*_ = 20°, ϕ = 0°), spectrometer and detector used.

**Figure 7:**
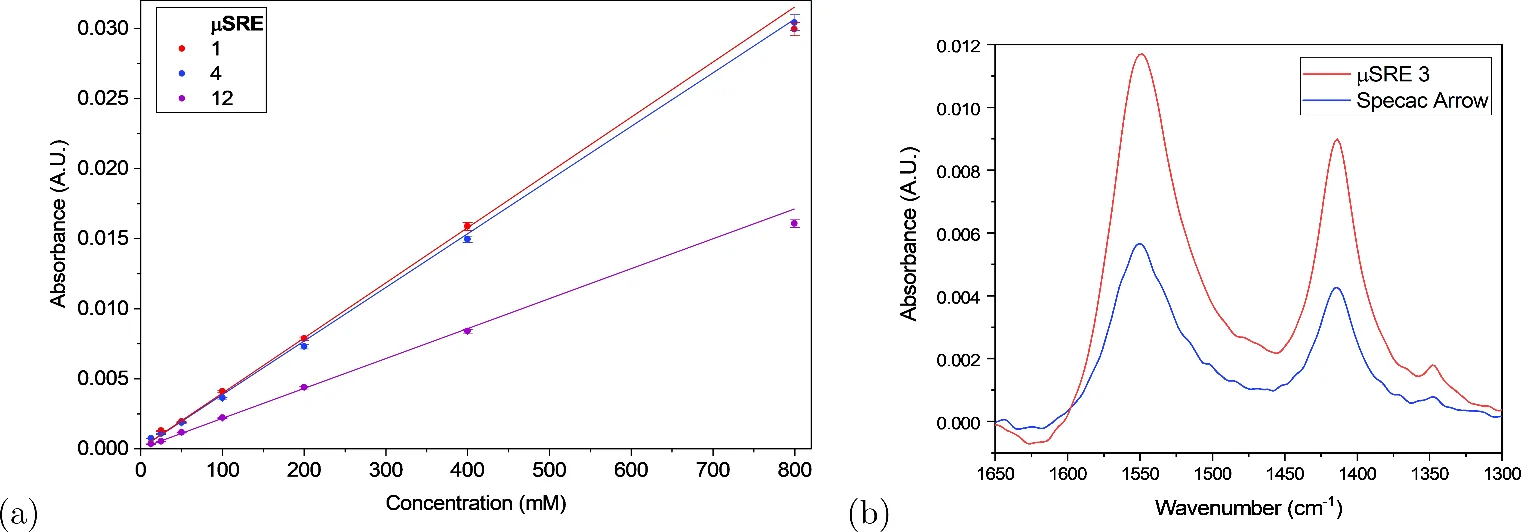
(a) Absorbance of serial dilutions of glucose measured on μSREs with differing prism sizes. (b) Spectra of 250 mM NaOAc backgrounded to water, measured on μSRE 3 and a Specac Arrow chip using the QUEST accessory.

IRUBIS LOD and LOQ far exceeds that of other devices, due to its superior sensitivity and RMS noise. The lower noise floor can be attributed to the absence of fringes in the IRUBIS spectrum noted by ref 9, resulting in greater throughput, which has an order of magnitude impact on the LOD and LOQ. Thus, minimization of fringes in these spectra has a noticeable impact on the performance of these devices. Dxcover and Specac chips show worse performance than IRUBIS; these commercially available chips use (100) Si, resulting in a larger TIR angle γ, and therefore a smaller evanescent field penetration depth, reducing sensitivity, while fringes are also observed in their spectra.

In comparison, μSRE 12 shows lower sensitivity than 1 and 4, which have larger values of *A*. However, μSRE 12 has lower noise, suggesting a greater throughput supported by the lack of strong fringes in its spectrum in Figure 3b. Thus, minimizing the fraction of the μSRE covered by prism roofs, and minimizing spectral fringes, is key to improving μSRE performance. Using the QUEST accessory, Specac Arrow chips were compared to μSRE 3. Figure 7b shows the 250 mM NaOAc spectra measured on each μSRE, where the μSRE we fabricated was cut to the same size as the Arrow chip. The calculated evanescent field penetration depth for μSRE 3 is 1.0 μm compared to 0.51 μm at 1550 cm^–1^ for Specac; indeed, μSRE 3 enables a spectrum to be recorded with absorbance double that for the commercially available Arrow chips.

Thus, to achieve optimal LOD, sensitivity, and minimal noise, the design of the μSRE must be carefully tuned to minimize spectral fringes due to interference between beams of differing path length by selecting appropriate prism dimensions. To minimize the risk of experimental error in any parameter causing fringes to occur, larger prisms are advantageous. To maximize sensitivity, the value of *A* should also be maximized, while a value of β = 54.7° maximizes evanescent field penetration depth.

## Conclusion

μSREs are a promising low-cost disposable replacement to conventional ATR crystals, with several commercially available options. However, these devices can suffer from fringes within their spectra, increasing noise and reducing the ability to extract quantitative data about a sample. This work proves these fringes are a result of an accrued phase difference between beams incident on the μSRE on the same prism face, but exiting the μSRE from separate prisms (or vice versa), resulting in interference. This can be avoided through careful design of the μSRE by optimizing prism period for given angles of incidence, with larger prisms providing less risk of experimental error introducing fringes into the spectrum. To maximize sensitivity, the choice of silicon wafer orientation must also be considered, with (110) wafers providing greater penetration depth, while the fraction of the μSRE surface covered by prism roofs should be minimized.

## Supplementary Material



## Notes

Full derivation of the calculation of the mathematical model for fringe period calculation, normalized FFTs of single beam spectra of μSRE 4, and unnormalized single-beam spectra of μSREs 1–15 without offset (PDF)
