# Supplementary material for: Optimization of Microstructured Silicon Attenuated Total Reflectance Fourier Transform Infrared Elements
Source: Anal Chem. 2026 Aug 27;98(35):25763–9. doi: 10.1021/acs.analchem.6c02804 (PMC13564101; doi:10.1021/acs.analchem.6c02804)
Supplement: Supplementary file 1 [file ac6c02804_si_001.pdf]

Supporting information: Optimization of Microstructured Silicon Attenuated Total Reflectance Fourier Transform Infrared Elements

Eleanor L. Osborne[a], Aneesh Vincent Veluthandath[a], Waseem Ahmed[a], and Ganapathy Senthil Murugan[a,*]

[a]Optoelectronics Research Centre, University of Southampton, University Road, Southampton, SO17 1BJ, United Kingdom
*Email: smg@orc.soton.ac.uk

Contents

Derivation of fringe period calculation **S2**

Fourier spectra **S5**

Single-beam spectra **S9**

Derivation of fringe period calculation

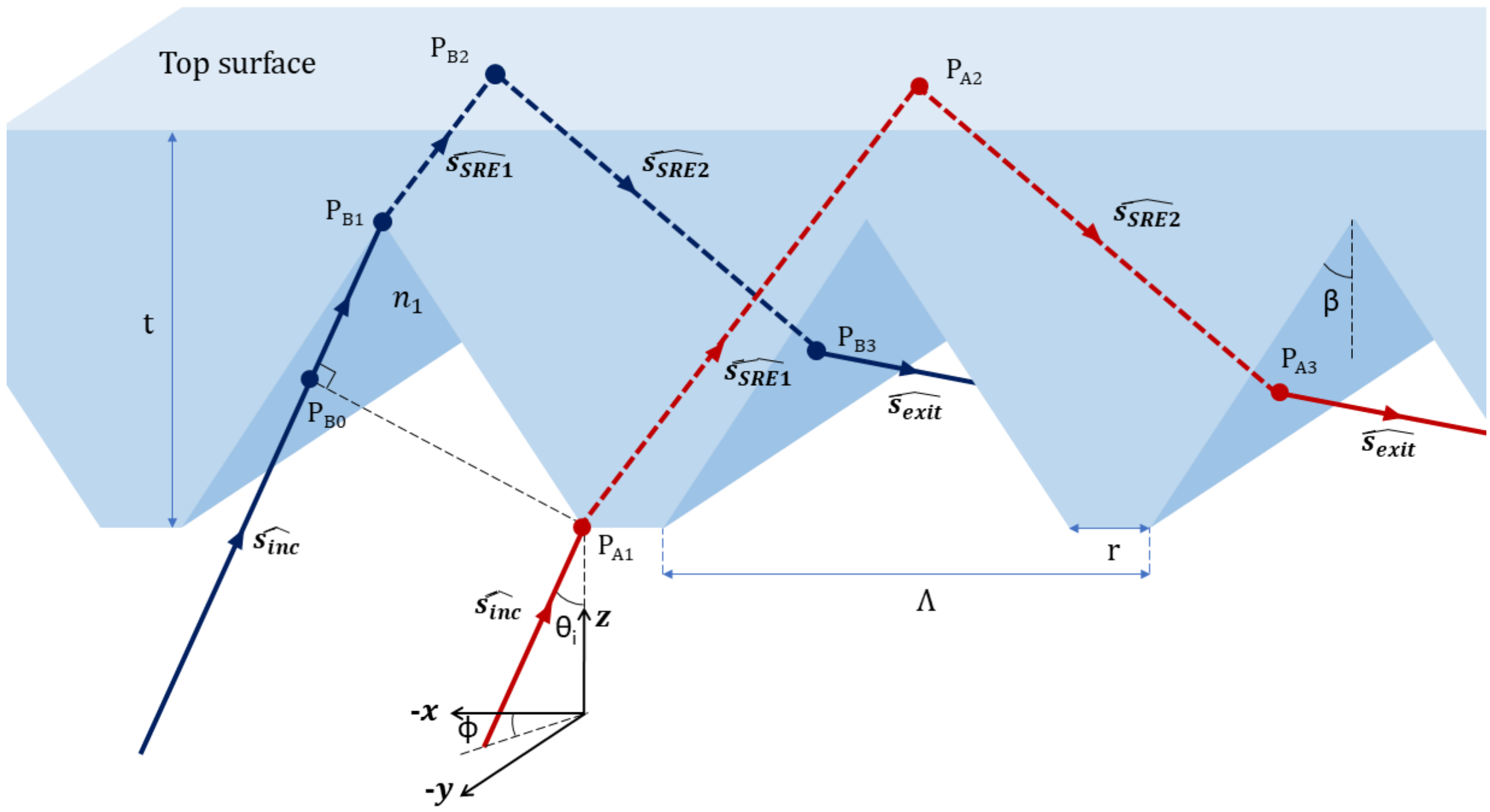

Figure S1: Schematic of a µSRE of thickness t with two beams (A and B) incident at points P_{A1} and P_{B1}, with incident azimuth angle ϕ and elevation angle θ_i. Both beams refract upon entry to the prism, totally internally reflect at the top surface at points P_{A2} and P_{B2}, then exit the µSRE at points P_{A3} and P_{B3}. Dashed lines represent the beam travelling through the µSRE.

Figure S1 shows two beams (A and B) that are part of a plane wave incident on a prism with unit vector $\widehat{\mathbf{s_{inc}}}$:

$$\widehat{\mathbf{s_{inc}}} = \begin{bmatrix} \sin(\theta_i)\sin(\phi) \\ \sin(\theta_i)\cos(\phi) \\ \cos(\theta_i) \end{bmatrix} \tag{1}$$

where θ_i is the elevation angle of incidence and ϕ is the azimuth angle of incidence. The unit normal vector to the prism surface is $\widehat{\mathbf{n}}$:

$$\widehat{\mathbf{n}} = \begin{bmatrix} -\cos(\beta) \\ 0 \\ -\sin(\beta) \end{bmatrix} \tag{2}$$

where β is the angle of the prism sidewall to the vertical.

The two rays are modelled, such that they intersect the prism at the two edges of the angled prism wall, but exit the µSRE through separate prisms, as shown in Figure S1. Whether this phenomenon occurs depends on the prism pitch Λ, and the incident angles of the beam.

The equation for the beam at any point is

$$\mathbf{r} = a\widehat{\mathbf{s}} + \mathbf{P} \tag{3}$$

where a is a scalar, $\widehat{\mathbf{s}}$ is the unit vector, and $\mathbf{P}$ is a position vector of a point P that the beam passes through.

To ensure the beams are in phase, they must have travelled the same distance from the source. Given that the beam is incident on the prism at an angle, with unit vector $\widehat{\mathbf{s_{inc}}}$, one edge of the prism will be hit first. To determine this, the incident angle in the x-z plane α is calculated as

$$\alpha = \arctan\left(\frac{\widehat{\mathbf{s_{inc}}}_x}{\widehat{\mathbf{s_{inc}}}_z}\right). \tag{4}$$

where $\widehat{\mathbf{s_{inc_x}}}$ and $\widehat{\mathbf{s_{inc_z}}}$ are the x and z components of $\widehat{\mathbf{s_{inc}}}$.

If $\alpha < \frac{\pi}{2} - \beta$, beam A is incident first, and vice-versa for $\alpha > \frac{\pi}{2} - \beta$. Therefore, the position of the other beam when the first beam hits the prism must be calculated. In the case shown, beam A hits the prism

first, so the position of beam B (P_{B0}) must be calculated. This is done by taking the dot product of the incident vector $\widehat{\mathbf{s_{inc}}}$, and the position vectors on the prism $\mathbf{P_{A1}}$ and $\mathbf{P_{B1}}$.

$$a_0 = (\mathbf{P_{A1}} - \mathbf{P_{B1}}) \cdot \widehat{\mathbf{s_{inc}}} \tag{5}$$

$$\mathbf{P_{B0}} = a_0\widehat{\mathbf{s_{inc}}} + \mathbf{P_{B1}} \tag{6}$$

The rays are then traced from points P_{A1} and P_{B1}, undergoing refraction to have unit vector $\widehat{\mathbf{s_{SRE1}}}$, calculated using the vector form of Snell's law [1]:

$$n_{air}(\widehat{\mathbf{s_{inc}}} \times \widehat{\mathbf{n}}) = n_{Si}(\widehat{\mathbf{s_{SRE1}}} \times \widehat{\mathbf{n}}) \tag{7}$$

The top surface of the µSRE has unit normal vector $\widehat{\mathbf{m}}$,

$$\widehat{\mathbf{m}} = \begin{bmatrix} 0 \\ 0 \\ 1 \end{bmatrix} \tag{8}$$

and equation

$$\widehat{\mathbf{m}} \cdot \mathbf{P_{top}} + b = 0 \tag{9}$$

where

$$\mathbf{P_{top}} = \begin{bmatrix} 0 \\ 0 \\ t \end{bmatrix} \tag{10}$$

and b = -t (the thickness of the Si chip).

The beams undergo total internal reflection at the top surface, having new vector $\widehat{\mathbf{s_{SRE2}}}$:

$$\widehat{\mathbf{s_{SRE2}}} = \widehat{\mathbf{s_{SRE1}}} - 2(\widehat{\mathbf{s_{SRE1}}} \cdot \widehat{\mathbf{m}})\widehat{\mathbf{m}} \tag{11}$$

The position vectors of the points at which the beams intersect the top surface of the µSRE is calculated using the equation:

$$a_1 = \frac{-(\widehat{\mathbf{m}} \cdot \mathbf{P_{A1,B1}}) + b}{\widehat{\mathbf{m}} \cdot \widehat{\mathbf{s_{SRE1}}}} \tag{12}$$

$$\mathbf{P_{A2,B2}} = a_1\widehat{\mathbf{s_{SRE1}}} + \mathbf{P_{A1,B1}} \tag{13}$$

This process is then repeated for intersection with the prisms on the output of the µSRE. $\widehat{\mathbf{n_r}}$ is the unit normal to the output prism face:

$$\widehat{\mathbf{n_r}} = \begin{bmatrix} \cos(\beta) \\ 0 \\ -\sin(\beta) \end{bmatrix}, \tag{14}$$

such that

$$a_2 = \frac{-(\widehat{\mathbf{n_r}} \cdot \mathbf{P_{A2,B2}}) + b}{\widehat{\mathbf{n_r}} \cdot \widehat{\mathbf{s_{SRE2}}}} \tag{15}$$

$$\mathbf{P_{A3,B3}} = a_2 \cdot \widehat{\mathbf{s_{SRE2}}} + \mathbf{P_{A2,B2}} \tag{16}$$

where the unit vector of the beam exiting the prism is calculated using Snell's law:

$$n_{Si}(\widehat{\mathbf{s_{SRE2}}} \times \widehat{\mathbf{n_r}}) = n_{air}.(\widehat{\mathbf{s_{exit}}} \times \widehat{\mathbf{n_r}}) \tag{17}$$

Finally, to find a point at which both beams have an equal distance to travel to the detector (and should therefore be in phase if constructive interference is to occur),

$$a_3 = (\mathbf{P_{A3}} - \mathbf{P_{B3}}) \cdot \widehat{\mathbf{s_{exit}}} \tag{18}$$

$$\mathbf{P_{B4}} = a_3\widehat{\mathbf{s_{exit}}} + \mathbf{P_{B3}}. \tag{19}$$

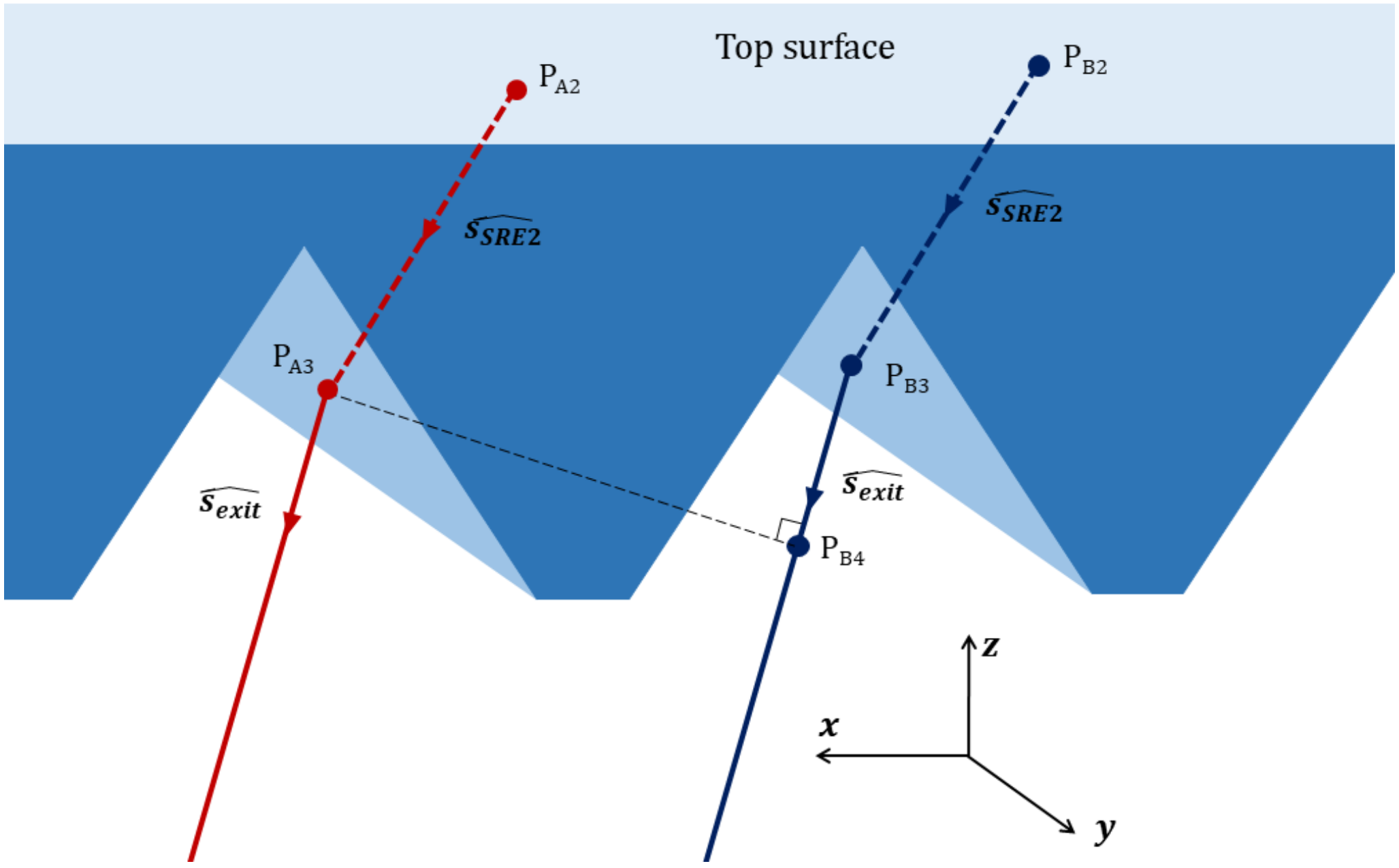

Figure S2: Beams A and B exiting the µSRE at points P_{A3} and P_{B3}. Dashed lines represent the beam travelling through the µSRE.

This is shown in Figure S2. Total distance travelled in the µSRE (d_{Si}) and air (d_{air}) for beams A and B are then calculated, and used to determine conditions in which constructive interference occurs in accordance with the relation:

$$m\lambda = n_{air}\Delta d_{air} + n_{Si}\Delta d_{Si} \tag{20}$$

Fringe period F_Λ is:

$$F_\Lambda = \frac{1}{n_{air}\Delta d_{air} + n_{Si}\Delta d_{Si}} \tag{21}$$

assuming the effect of dispersion is negligible.

Fourier spectra

Figures S3, S4, and S5 show fast Fourier transforms discussed in the main text. The peaks show broadening compared to the mathematically predicted single fringe period given by the derivation in the section 'Derivation of fringe period calculation'. This is attributed to the effect of dispersion across the full spectral range, resulting in small changes in fringe period across the spectral range. Furthermore, the convergent beam of the spectrometer will result in a distribution of incident angles around the expected value, further resulting in broadening. In Figure S4, it can be seen that there is a shift to smaller fringe periods with greater elevation angle of incidence θ_i. Graphs for $\theta_i = 30°$ and $\theta_i = 50°$ do not show clear peaks at any given fringe period, in agreement with their single beam spectra in Figure 5a in the main text which show only weak fringes. This also supports the mathematically calculated fringe periods shown in the main text that suggest there are incident angles at which fringes will not be produced in the spectra, as all the beam that is incident on one prism face will exit the µSRE through another single prism face also. In contrast, Figure S5 shows an increase in fringe period with increasing azimuthal angle ϕ.

Figure S3 shows fast Fourier transform spectra for each of the fifteen µSREs tested. µSREs 1-7 show more clear peaks corresponding to theoretically predicted fringe periods. However, for µSREs with large prism pitch, the fringe period becomes difficult to discern from the Fourier spectra. For µSREs 1 and 2, with very small pitch, show second smaller fringe periods in addition to the strongest peak, which is also visible in their spectra in Figure 3b in the main text at smaller wavenumbers. These fringes are an integer fraction of the first order fringe, as expected.

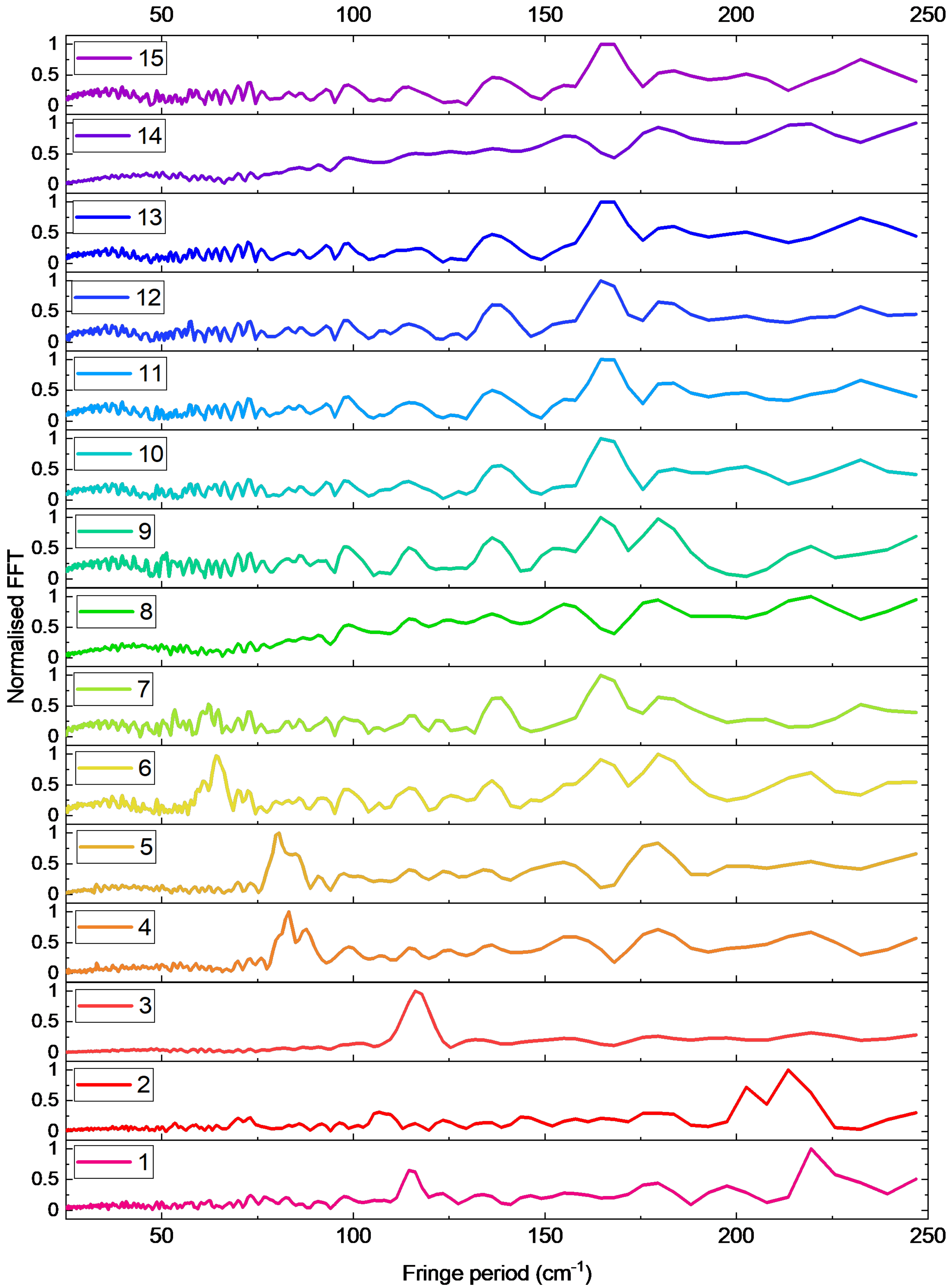

Figure S3: Normalised fast Fourier transforms of single beam spectra (air) using different µSREs with incident angles $\theta_i = 40°$ and $\phi = 0°$.

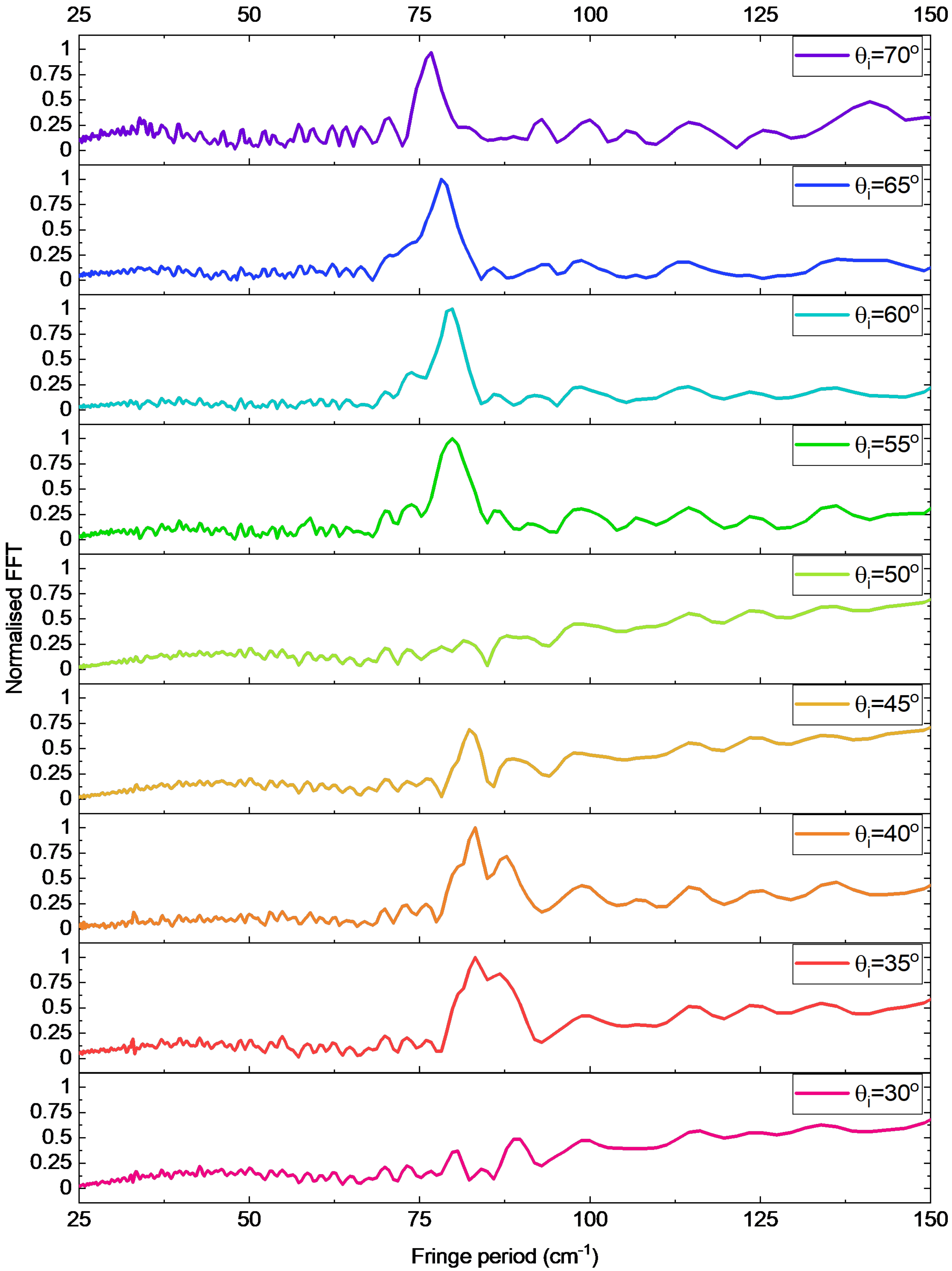

Figure S4: Normalised fast Fourier transforms of single beam spectra (air) using µSRE 4 with incident angle $\phi = 0°$ and varying θ_i.

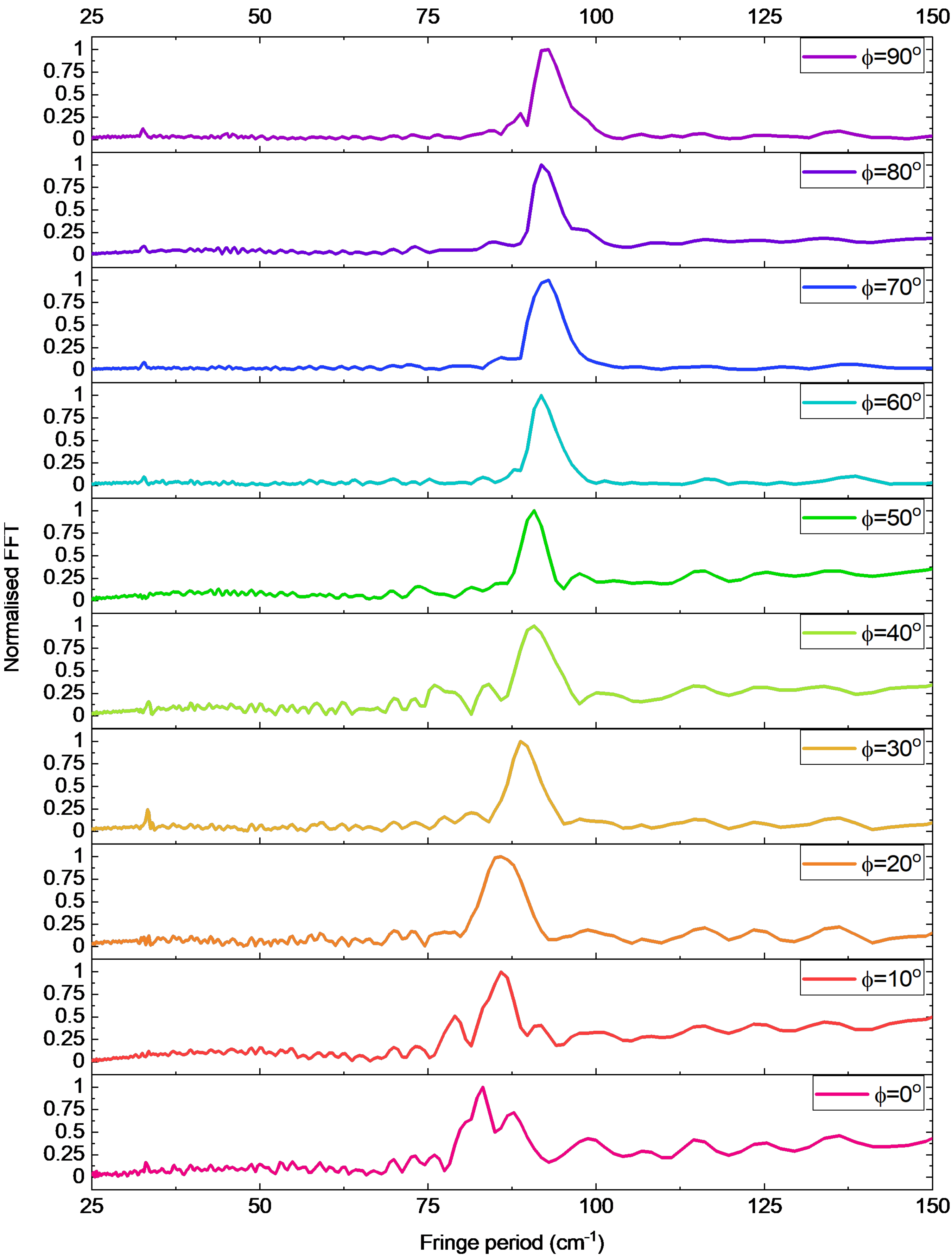

Figure S5: Normalised fast Fourier transforms of single beam spectra (air) using µSRE 4 with incident angle $\theta_i = 40°$ and varying ϕ.

Single-beam spectra

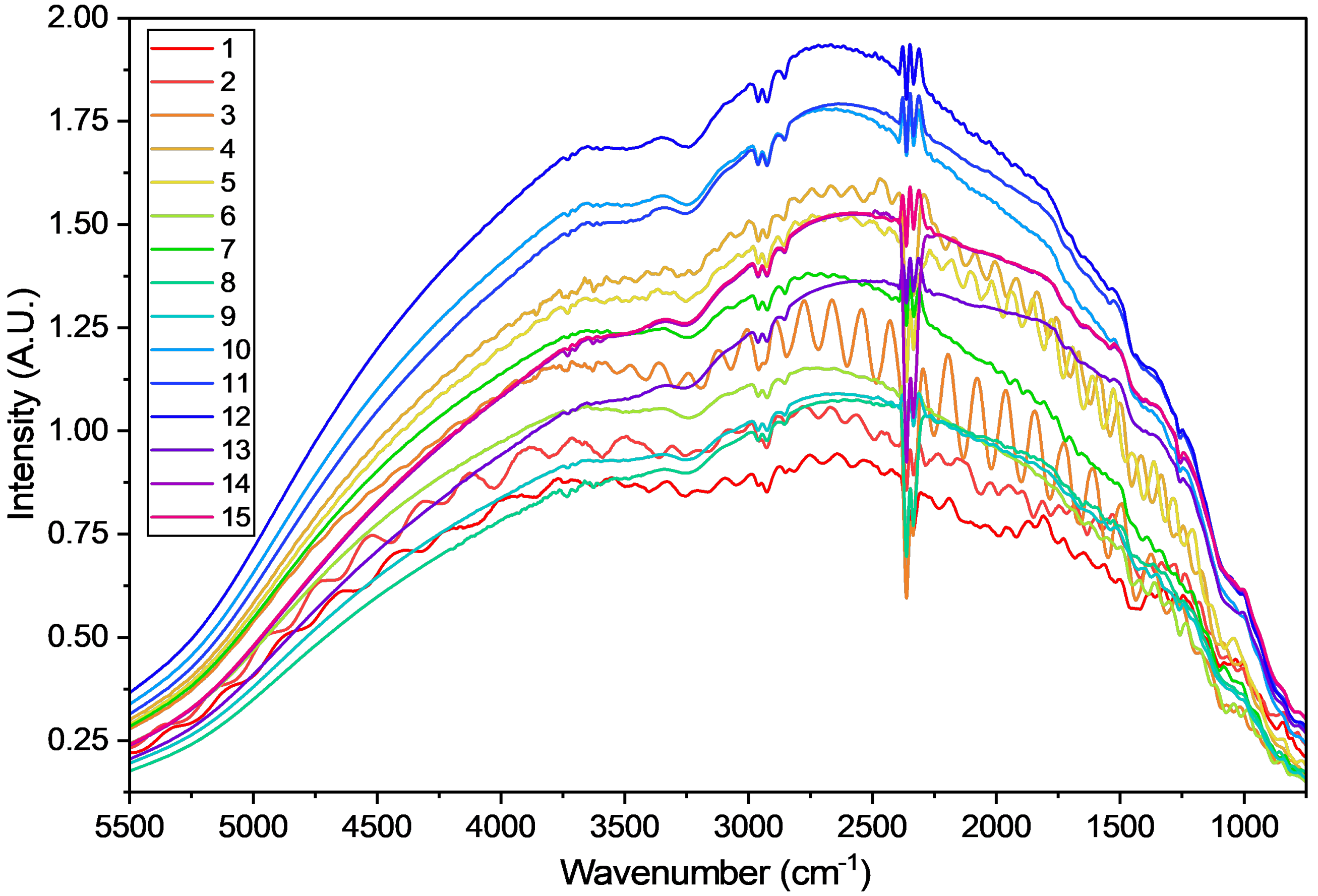

Figure S6: Single beam spectra of µSREs 1-15 without normalisation or offset.

References

1. Kingslake, R. & B. Johnson, R. in *Lens Design Fundamentals (Second Edition)* (eds Kingslake, R. & B. Johnson, R.) Second Edition, 25–49 (Academic Press, Boston, 2010). ISBN: 978-0-12-374301-5. https://www.sciencedirect.com/science/article/pii/B9780123743015000061.
